# Health workforce perceptions on telehealth augmentation opportunities

**DOI:** 10.1186/s12913-023-09174-4

**Published:** 2023-02-21

**Authors:** L. T. Thomas, C. M. Y. Lee, K. McClelland, G. Nunis, S. Robinson, R. Norman

**Affiliations:** 1 School of Population Health, Curtin University, Perth, WA Australia; 2 Government of Western Australia Department of Health, Perth, WA Australia; 3 WA Primary Health Alliance, Perth, WA Australia; 4 Deakin University, Melbourne, VIC Australia

**Keywords:** Telehealth, Service improvement, Qualitative research

## Abstract

**Background:**

The availability and use of telehealth to support health care access from a distance has expanded in response to the COVID-19 pandemic. Telehealth services have supported regional and remote health care access for many years and could be augmented to improve health care accessibility, acceptability and overall experiences for both consumers and clinicians. This study aimed to explore health workforce representatives’ needs and expectations to move beyond existing telehealth models and plan for the future of virtual care.

**Methods:**

To inform recommendations for augmentation, semi-structured focus group discussions were held (November–December 2021). Health workforce representatives with experience in health care delivery via telehealth across country Western Australia were approached and invited to join a discussion.

**Results:**

Focus group participants included 53 health workforce representatives, with between two and eight participants per discussion. In total, 12 focus groups were conducted: seven were specific to regions, three with staff in centralised roles, and two with a mixture of participants from regional and central roles. Findings identified four key areas for telehealth augmentation: improvements required to existing service practice and processes; equity and access considerations; health workforce-focussed opportunities; and consumer-focussed opportunities.

**Conclusions:**

Following the onset of the COVID-19 pandemic and the rapid increase in health services delivered via telehealth modalities, it is timely to explore opportunities to augment pre-existing models of care. Workforce representatives consulted in this study suggested modifications to existing process and practice that would improve the current models of care, and recommendations on ways to improve clinician and consumer experiences with telehealth. Improving experiences with virtual delivery of health care is likely to support continued use and acceptance of this modality in health care delivery.

## Background

The COVID-19 pandemic significantly improved the availability of telehealth and virtual care modalities in the health care system [1]. This trend is likely to generalise internationally but is particularly relevant in a setting where a significant proportion of the population experiences geographical (and in some cases digital) isolation, necessitating innovative solutions to provide equitable and effective care for regional, rural and remote residents. Western Australia is an example of such a setting and accounts for 34.3% of the land area of all Australia [2] with the majority of its 2.7 million residents living in its capital city, Perth [3]. The regional, rural and remote regions of Western Australia have the second lowest population density of all states and territories and span more than 2.5 million square kilometres.

Telehealth involves the use of information and telecommunication techniques, such as telephone and video conferencing, to provide health and medical consultation at a distance [4]. Between 2012 and 2021, there were 185,511 outpatient appointments delivered by telehealth (video) to country residents in Western Australia. In the 2021–22 financial year, more than 43,000 outpatient telehealth (video) appointments were provided to regional patients, demonstrating a 833% growth since 2012. Attending outpatient appointments by telehealth during the 2021–22 financial year saved Western Australian patients 46 million kilometres of travel. With the advent of technology, additional virtual care technologies (e.g. remote monitoring equipment, smart phone apps) can be used to support more traditional telehealth modalities to deliver health care across an increasing range of disciplines [5].

In response to the rise in virtually delivered health care, healthcare workers were rapidly required to upskill in online appointment etiquette, online assessment implementation and the intricacies of various software applications [6]. Moreover, they needed to have an understanding of information and technology requirements and trouble-shooting options to support patients to effectively participate in virtual appointments [7, 8]. In some instances, moving to online delivery mechanisms arose overnight, with limited opportunity for the required workforce training [9].

Telehealth can provide many benefits for patients [10–12], most notably the potential to reduce travel time and cost incurred by regional patients [1, 13, 14] and the increased access to service providers, in particular specialised care which is often clustered in metropolitan centres [15]. Perceived barriers to use of telehealth, from a patient perspective, include a lack of digital literacy and access, uncertainty around the quality of care delivered via virtual care modes, privacy concerns and preferences for in-person care [7, 14, 16]. Clinicians play a key role in navigating the implementation of virtually delivered health care by supporting patient needs and expectations through their ability to form relationships and trust, and educate through care provision [4].

Clinically, telehealth benefits include enabling remote triage of patients, rapid access to information, and remote diagnosis, care and patient management [4]. Evidence suggests, however, that many clinicians feel conflicted regarding their ability to deliver health care via virtual modes and the translation of care delivered via these modalities into positive patient outcomes [4, 17]. Challenges also arise in the integration of technology with current health systems to support adaptation of existing in-person clinical services to virtual delivery modes [9]. Barriers to using telehealth and other virtual modalities to deliver health care include: lack of confidence in delivery of care via telehealth, lack of digital literacy skills, preference for hands on/in-person care, internet quality and system barriers such as financial and policy restrictions [10, 14]. Limited evidence exists from the workforce perspective on what the workforce needs to support virtual delivery of health care, and make this modality a complementary part of care provision, rather than just the back-up option.

This study sought to explore the needs and expectations of regional and metropolitan health workforce representatives, to move beyond existing telehealth models and plan for the future of virtual care. This work centred on Western Australia, but is likely to generalise to other settings, particularly those high-income countries with significant regional and rural populations.

## Methods

### Participants

The project was developed through a Project Working Group, comprised of representatives from research, and key stakeholders involved in service design and delivery. These included the Western Australian Country Health Service (WACHS), the Western Australian Department of Health, the Western Australian Primary Health Alliance (WAPHA), Curtin University and the Digital Health Collaborative Research Centre (DHCRC). Working Group members were asked to nominate health workforce representatives with experience in health care delivery via telehealth and virtual care modalities across country Western Australia, and also those in central office roles. A database of 48 regional and 32 central office staff members was compiled. An email was sent to all listed representatives inviting them to join either an in-person or online, group or individual discussion about their experience of telehealth and virtual care, and their needs and expectations for this mode of health care delivery. Snowball sampling was accepted whereby individuals were permitted to share the email with colleagues they thought would be able to contribute to the discussion. Consumer input was also sought and will be reported separately.

### Data collection

Semi-structured group and individual discussions were conducted with all participants. Eight discussions were held solely online while three were in-person and one included a mix of online and in-person participants. Group discussions ranged in size from two to eight participants; with each WACHS region being represented in the data. Discussions ranged in duration from 66 to 102 min and were conducted in November and December 2021 by three Curtin University researchers (LT, RN, SR). An observer (CL) was present in all discussions except one in-person meeting (due to logistical reasons), in which the facilitator (LT) took notes.

### Instrumentation

The discussion guide was developed following a review of literature exploring clinicians’ experiences with telehealth and endorsed by the Working Group before being finalised and applied in the discussions. Questions pertained to workforce representatives’ positive and negative experiences with virtual modalities, an ideal-world vision for the future of virtual care and steps required to move from the current experience to the ideal-world vision (Table 1). The notes compiled by the observers were reviewed alongside discussion transcripts, providing insight into participant engagement in the discussion, and non-verbal cues. It is not expected that the change in observer impacted interpretation of participants’ responses.

**Table 1:** Key questions guiding discussions

1. Tell me about when you see telehealth and virtual care working well for country patients.
2. Tell me about when you see telehealth and virtual care not working well for country patients.
3. What do we need to change or add to the way telehealth and virtual care is currently delivered to improve the experience of telehealth and virtual care for the provider and consumers?
4. What needs to be implemented to continue to support the effective use of telehealth and virtual care?
5. What does the future of telehealth and virtual care look like for you?

### Data analysis

All interviews and group discussions were audio recorded and transcribed verbatim with identifying information removed. Thematic analysis followed six steps as outlined by Braun and Clark [18]. The main facilitator (LT) repeatedly read the transcripts for familiarisation. Initial codes were identified under the five main questions guiding the discussion. The first author collated codes into potential themes, reviewing and recoding until a pattern emerged and categories were developed. The research team reviewed the transcripts and codes to confirm the overarching categories and main themes established.

## Results

Participants were 32 regional and 18 central representatives employed by Western Australian Health (government department), plus three non-government organisation representatives, encompassing administrative, clinical, technical and policy roles. Four key themes were identified from the data: (a) service improvement recommendations; (b) equity and access considerations; (c) health workforce-focussed augmentation; and (d) consumer-focussed augmentation.

### Existing service improvement recommendations

Across group discussions, the culture where telehealth is viewed as a *“back-up therapy option”* [Participant 401], rather than as a mainstream complementary and viable delivery option was seen as a barrier to increased provision by clinicians and uptake by patients.

*“What I would just really like [is] for just the culture to change so that it’s a valid treatment option in itself. Like, which is the best for you and have it just be sort of a valid option?”* [Participant 401]

Many participants provided suggestions on how to improve the current system to enhance clinician and patient experience. There was a perception among many participants that digital information sharing and communication requirements could be streamlined to reduce “*the administrative burden behind it—we do need to have these assessment forms and our handouts and everything adapted into an electronic version that we can fill in and send … to make it more efficient.”* [Participant 604].

This would also assist to increase access between and within Health Service Providers and benefit patient experiences, as noted by Participant 703: *“… they [digital health technologies] can already, you know, pick up our ECG's [electrocardiographs] … they can't pick up our … blood results. At the moment, we're doing the old fashioned print off the bit of paper, photocopy it, scan it and send it down … in future there will be more … of the machines that we use, that they can pick up, which is all going to be time saving for us.”*

Information sharing was recommended to include the regional context in which patients lived to address a perceived lack of understanding of regional issues among metropolitan clinicians. As one respondent noted, *“I don't think Metro really understands, as much as you try and explain to them, that we live in a region that patients are very transient, we can confirm an appointment, and it's for the next day, and then all sudden they're not there and they don't turn up, and …we've been trying to find patient.”* [Participant 101]

This lack of understanding was seen to have an impact on patient access to telehealth, and on the implementation of care plans following treatment.

*“A lot of the telehealth that we've traditionally up until now received into our region has been from the metro sites, and I think there still needs to be greater awareness at the metro sites about the resource pressures in our regional sites.” *[Participant 202]

*“From a metropolitan perspective, and there's very little understanding of regional patients of actual distances, not everyone can go five minutes down the road and access an X ray.”* [Participant 601]

Participants associated opportunities to practice (in a test situation) care delivery via telehealth with increased clinician confidence. This, coupled with a strong, telehealth-specific support team (providing information technology and technical support) was viewed as essential to increase clinician enthusiasm to use virtual care modalities.

*“Having like a practice run beforehand and having that as something that can be captured and acknowledged as part of your role.”* [Participant 101]

*“If the staff aren't confident with the equipment and how to use it, then how on Earth are they going to impact that onto a patient? And then they're not going to because they've not got confidence, they're not showing that, so the patient is going, ‘well, I can't do it either’.”* [Participant 701]

At the patient interface, workforce representatives noted capturing telehealth preferences would be a useful addition to existing electronic patient records, and recommended an online booking system would empower consumers to request telehealth delivery modes for their health care.

### Equity and access considerations

Central to many recommendations raised by workforce representatives was the need for access to healthcare to remain accessible and equitable. This includes access to hardware, software, telephone credit and data required to participate in digital health appointments. Existing systems offering a library of hardware to loan to patients was reported as being successful in increasing access for those patients who did not have their own devices to use; however limited supplies affected the capacity of reach.

*“We do have a supply of iPads that we can loan out to those clients [who do not have their own device], and in the event that all those iPads are already out on loan, we would try, and hopefully they're close to, the local health service.”* [Participant 402]

As with any technology, access to reliable connectivity was essential to effective and confidential participation in telehealth. In the absence of their own devices, where patients could access centralised digital infrastructure, workforce participants noted the inclusion of a telehealth coach/supporter would improve patients’ telehealth confidence and experience. In areas where centralised telehealth facilities were unavailable, or too distant, participants recommended *“use [of] the AMS [Aboriginal Medical Service] services wherever they are [place names removed], or alternatively, identify areas within the shires, or at other authorities, the mining industry, for example, where they can make facilities available for telehealth consultation to happen there,”* [Participant 504] to support access to strong internet connections.

Partnerships with community-based organisations were also considered essential to ensure equity and access, particularly for Aboriginal communities.

### Health workforce-focussed augmentation

Participants reported modifications that would enable more effective delivery of health care via telehealth in the more-immediate term and as part of a longer-term approach to increased telehealth delivery. In regards to more immediate responses, participants noted the rapid response to mobilising telehealth delivery at the onset of the pandemic, combined with hardware supply availability had resulted in implementation of resources, that may not have been the preferred choice. As an example, *“having better equipment, like we don't have noise cancelling headphones. We don't have microphones that can block out external noise, so if we had better quality equipment in addition to better quality spaces where we can … actually do dedicated telehealth appointments, in a quiet space,”* [Participant 604], this would have a positive impact on both patient confidentiality and clinician distraction*.*

A telehealth community-of-practice, both general and discipline specific, was reported as an opportunity to share resources and training, successes, and support. This, paired with clinical practice guidelines outlining how to do previously in-person assessments via digital technologies would increase clinician’s capacity to deliver effective health care.

*“I know there's lots of work in this space … tools for clinicians that are like assessment tools, clinical assessment tools that are suitable for telehealth application. I know that there is more and more becoming available … I think that was part of the exhaustion and fatigue that we were experiencing was that we were trying to like … you've got this meta cognition thing going on. Where you're trying to see your client, trying to help them to feel relaxed and comfortable about what they're doing, but at the same time, thinking how the hell am I adapting this thing to this platform so it's just appropriate tools as well.” *[Participant 602]

The overriding improvement raised at all discussions was the need for integrated systems ensuring ease of access to patients’ medical records. This encompassed a common platform to which multiple existing programs would connect.

*“There were conflicting telehealth appointments during COVID because we’d have all the ones coming from the metro ones that we normally had, but then we had all these inter-regional ones as well. So the room would be double-booked because different people were booking it and there was no integration even within the telehealth system… that might have changed now but it just showed to me that everyone was working on their own service and not that sort of bigger picture.” *[Participant 302]

*“Better integration… having that documentation more seamlessly you know, as well as availability of results through other systems.”* [Participant 601]

Improved pathways for image sharing which also communicated into the same common platform, and provided better resolution and magnification for more accurate image capturing would enhance clinician experience and reduce administrative time.

*“I would love there to be one electronic medical record for the patient. Like that would have to be one of the most frustrating things coming to [this state], and there not be one system for every single health professional is documenting into regarding that client. We do a lot of case management and care coordination in our role and I would love to be able to see… that my patient is attending their specialist appointment… when they last attended the pharmacy and filled a script, 'cause that would tell me if they’re taking their tablets…, that they had their blood tests.”* [Participant 402]

When considering future planning and design which supports health care delivery via telehealth, participants reflected on the need to consider adequate *“lighting, you know, natural light”* [Participant 604] and workstation configuration (including private telehealth consultation rooms for clinicians, intuitive cameras to follow clinicians as they move to provide demonstrations for patients and standing desks). As stated by one participant, *“we do need to look after our clinicians a little bit more so, by having standing desks, so you've got the opportunity to stand up or sit down as you need to. And maybe having different interfaces where it's just, you know you don't have to try so hard to be engaging in the client. All goes into fatigue management and then overall clinician wellbeing and burnout.” *[Participant 602]

### Consumer-focussed augmentation

Health consumers can provide design and service improvements based on their experience and need to be key partners in co-design. From health workforce perspectives, however, consumer experiences would be enhanced through augmentation to the patient appointment experience, digital equity and access, and supporting convenient access to care details. First, consideration was given as to how to improve the existing experience from various locations/modalities as best fit for the appointment, *“currently they'll see, say [sic] receptionist and the specialist area. They just take them to the [telehealth] room and put them in there and say thank you very much. There's no one there …*.” [Participant 301]

Improving the patient experience may include supporting those patients who attend health facilities for their telehealth needs – including a receptionist to ‘meet and greet’ patients. One site discussed a role for a telehealth usher, *“so that he can say, you know, ‘Hi, how you going today’ and make the people feel comfortable because a huge percentage of ours [clients] are Aboriginal people, so feeling uncomfortable in the environment. You know, have you had a telehealth appointment before? Would you like an ALO [Aboriginal Liaison Officer] or an interpreter in with you? Someone basically to make them feel more secure in the appointment … Or say to them, ‘This is just like a normal doctor’s appointment, except the Doctor will be on the screen. You can still ask him all the same questions … this is secure, nobody else is listening in or watching him.’ Just to reassure people or see if they do need support.”* [Participant 301]

The creation of “a *virtual check-in [waiting room] and then the clinics can communicate directly with the patients to say, ‘Look, the doctor’s running 10 min late’, or ‘You're the next patient’, the same way that patients sitting in a waiting room are communicated [with]”* [Participant 202] would enhance patient experiences by connecting them with the service provider, and reassure them when appointments do not occur as scheduled.

Workforce representatives commented on the ability to improve digital equity and access through use of a free-call and/or free-data phone number (as exists in telephone systems in Australia with 1800 phone numbers). This system could exist to provide data/credit to patients to use on telehealth appointments when this would otherwise be unavailable to them.

*“Lots of the clients that I see don't have a fixed sort of mobile number or phone that they have. It kind of gets shared within their family group or may change and we may just you know, lose the patient because of that. And also, if they don't have credit or they don’t have data, so I think that's a challenge.” *[Participant 101]

*“It comes back to access to data and all of those sorts of things so that changes with certain populations within the [region], particularly those of the lower socio economic groups. So it's very complex as to how we ... You've got to think of all of these things in order to create a service that will work. And be equitable for everyone.” *[Participant 702]

*“I do think better access to Internet services in remote communities is a key, 'cause then you could do more telehealth in home; or you know even like community access to wireless Internet that's free, so that if you have got a smart phone or you can borrow a phone then you can have your telehealth appointment if you don't need support services around it.”* [Participant 302]

Finally, participants reported a one-stop portal (or app) which contained telehealth appointment details, appointment links and reminders would improve patients’ sense of control over their telehealth experience.

Futuristic ideas provided in several group discussions centred around improving patients’ access to telehealth infrastructure.

*“The ability that a TV could be enabled to be a camera as well and you could remotely call the patient through their TV because … a lot of our patients are sitting in front of their TV at home, they're not answering their phone all the time.”* [Participant 402]

*“Something like … the old public telephone… it could be in a remote community that anyone can access … they can walk there, drive there, hop, skip and jump there. They can log in. They can use this thing in a little confidential pod like a public telephone, you know, so it's not reliant on people to also have all of the equipment, technology, access, Internet, etc., like back in the day.”* [Participant 503]

## Discussion

This study explored health workforce representatives’ perceptions on how to augment telehealth and other virtual modalities to better respond to the increase in health care delivered by these means to support regional and remote patients. Participants’ responses were focussed on improvements needed to existing practice and processes, suggesting these may need to be addressed before additional technology supports may need to be considered. While service delivery via telehealth is well advanced in Western Australia [13], and a commitment to support this mode of service delivery exists [19], the rapid advance of digital technologies suggests regular revision is warranted. In this regard, participants discussed existing service improvement recommendations, equity and access considerations and innovations that could improve clinician and patient telehealth capacity and experiences.

Australia’s National Digital Health strategy [20] seeks to establish a sustainable health system that constantly improves, with two strategic priorities directly relating to telehealth and virtual care. These focus on digitally enabled models of care and a digitally-confident workforce which combine to realise benefits for patients. In the Western Australian context, telehealth models for regional and remote residents are well advanced [13], this modality is an organisational priority for all health service providers and it is a stated health reform priority [19]. Hence, there is great interest in the use of health care delivered virtually.

Since telehealth began in Western Australia (about 25 years ago) [15], improvements have been made to ensure telehealth meets the needs and expectations of clinicians and patients alike. Uptake has been differential across regions, reflecting both responses to varying public health measures and access issues in rural and remote areas. The COVID-19 pandemic created a rapid and urgent need to transition many health appointments online [6], especially when regions were locked down and unnecessary travel was restricted. Clinicians and patients needed to upskill in telehealth modalities, infrastructure was required to support modified health care delivery and workflow adjustments were made to reflect these changes. When such a rapid response is needed, time is not available to reflect on how best to design and support clinical care delivery via these digital mechanisms.

Two years on from the onset of the pandemic, research has identified barriers and enablers to telehealth from a patient and clinician perspective [16, 21]. While much attention has been directed at these aspects of virtual care operations, limited research has explored what is needed to take virtual care modalities to the next level, and recognise telehealth as a legitimate mode of care delivery, when appropriate for patient and clinical requirements. Contexts are an important consideration in maintaining clinical service delivery via telehealth modalities, with telehealth practice legitimisation, confidence development, relationship management and resource provision being key mechanisms to support telehealth delivery [22]. These mechanisms are supported by findings in this research. Virtual delivery of care is recognisably different to in-person care in regards to the infrastructure requirements, scheduling capacities and support needs [21]. Clinicians provide a unique perspective on what is needed to move beyond traditional telehealth provision, being at the coalface of care delivery.

### Improve clinician and patient telehealth capacity and experiences

While not all consultations are appropriate to be delivered via telehealth modalities, nor all patients appropriate for care via telehealth; when telehealth can be utilised, it provides benefits for patients, clinicians and the health system [1, 10–12, 14, 15]. Ensuring positive patient and clinician experience of health care is paramount to improved patient outcomes.

Similar to Thomas et al. [9], this research identified a lack of clinician confidence and experience in delivering health care via telehealth modalities contributed anecdotally to lower uptake and continuation of care in this mode. While initial reluctance may be explained due to a hesitancy towards change away from ‘historical’ processes [23, 24], this research identifies some of the solutions required to influence increased and maintained uptake of virtual care modalities into the future.

From the system perspective, embedding change management strategies in health care workflow systems can assist in changing the culture around telehealth modality use. Implementing flexible performance indicators and targets for telehealth delivery [9, 21], and ensuring metropolitan clinicians are fully cognisant of the impact on patients that attending metropolitan-located appointments has [21], may assist in increasing clinician’s consideration of the appropriateness of appointments for virtual delivery modes, while balancing patient preferences [6, 25] and clinical requirements. Additional training in digital technologies relevant to telehealth may also improve clinician confidence and capacity [26]. A review of funding models associated with telehealth care provision, including the ability to claim for clinician attendance at both ends of the appointment (service provider as well as patient supporter) would likely contribute to an increase in the perceived value of clinician time and collaborative care [9, 27].

### Existing service improvement recommendations

Strategies which support the augmentation of existing telehealth practice include: creating supportive physical (e.g. adequate lighting, ergonomic consideration, quiet space) [6] and socio-emotional environments (e.g. communities of practice, practice opportunities in test scenarios) [6, 9] for the delivery of effective telecare; implementing effective policies that encourage telehealth modalities, where and when appropriate [1, 8]; and reorienting health services to support the positioning of telehealth modalities on an equal level to in-person and at-home health care, when appropriate considering the patient and clinical requirements.

In regards to the health workforce, many barriers to telehealth implementation relate to clinician preference and skills [27]. Thus, a gradual, staged approach providing immersive, practical opportunities for clinicians to experience and reflect on care delivery through digital modalities is likely to reduce barriers to use and support integration into practice [28]. The opportunity to practice telehealth and virtual care delivery modes prior to using them in a patient-facing situation would provide greater clinician confidence in employing these modalities in clinical practice [6]. Including training opportunities in the undergraduate environment would build workplace readiness skills required to provide care via telehealth upon entering the workforce [9]. Observing, modelling and imitating (as per Bandura’s Social Cognitive Theory [29]) virtual delivery of care provides reinforcement within a safe environment. Successful experiences lead to increased self-efficacy, and consequently behavioural adaptations.

Access to telehealth-specific information technology support centres would enhance workforce confidence in delivering care via digital modalities. This would assist by removing workforce concerns that technology challenges would erode patient trust in the clinician’s ability to provide effective care. Integrated information systems that communicate with each other within and between sites not only improves clinicians’ ability to provide care, but also reduces administrative burden associated with moving between digital applications, copying details, and restricted access issues [8, 21]. Some respondents noted the need to integrate telehealth-enabled care with the electronic health record and explore the interoperability of the health data. This could enable integration of different data sources including laboratory or radiology results and images.

### Equity and access considerations

Health workforce participants in this study acknowledged the role of patients’ digital and health literacy skills in effective virtual care experiences. Some participants noted differences in regional, cultural and other social determinants that relate to health equity and use of telehealth digital technologies. Empowering patients to note a preference for and request appointments via telehealth modalities be considered [9], and supporting improved digital literacy skills to participate effectively in telehealth appointments would activate patient engagement in digital health activity. These recommendations align with the three horizons to Australia’s reimagined health system presented in the recent white paper – Australia’s health reimagined: connected consumer, empowered consumer, and confident consumer [30]. Ensuring equitable access to healthcare must remain at the forefront of telehealth augmentation. Creative solutions may be required to support patient access to adequate internet connections. Participants in this research suggested this could occur through telehealth satellite centres at community resource centres, shire offices and mine site camps.

Underpinning the effective, equitable delivery of health care via telehealth and virtual care technologies is adequate access to internet connectivity. While the issue of poor connectivity in regional areas is well known [6, 31] and a long-standing barrier to effective telehealth delivery, so too is the ability to pay for telehealth access (phone call and data use costs) by our most vulnerable populations. Schemes which provide free-call access to telehealth appointments (similar to the 1800 phone number scheme in Australia), or which provide access to unused data, such as the Optus data donation scheme [32], would increase opportunities for vulnerable community members to access data required for digital appointments.

A strength of this study is the strong representation of regional workforce across Western Australia. Group discussions were largely held online to provide access to participants across regions with great geographical distance. The telehealth-related experiences, needs and expectation of clinicians, coordinators and administrative staff were captured, providing a broad understanding of how best to augment existing service provision. Participants were recruited from a list of workforce representatives recommended by the Working Group, however snowball sampling methods enabled expansion of recruitment to others who may have had relevant telehealth experiences and ideas. The findings of this study present the perspective of regional workforce representatives. Exploring the perspectives of metro-based clinicians who provide care via telehealth modalities into regional areas would improve our understanding of this experience.

## Conclusion

Rapid responses to move to increased telehealth and other virtual modes of health care provision were implemented as a result of COVID-19 pandemic-related public health restrictions. While telehealth modalities have been used for some time to support regional and rural resident access to a range of health services, this increase in virtual delivery contributed to service continuity during this period and maintained the safety of the health workforce and community members in the context of COVID-19. With the advent of time, comes the opportunity to reflect on how we can improve on these rapid responses to provide high quality care, and optimise both patient and health workforce experiences virtual delivery modes. Recommendations from the coalface, from those staff delivering digital health care to patients, provide a unique perspective on what is needed to improve the efficacy of existing models of care, and to maintain workforce satisfaction with care delivery. The recommendations arising from this research present an opportunity to move beyond traditional telehealth models and embrace new approaches to health care provision.

## Data Availability

This is a qualitative study. All data generated or analysed are included in this published article.
